# Overwhelming rapid metabolic and structural response to apatinib in radioiodine refractory differentiated thyroid cancer

**DOI:** 10.18632/oncotarget.15036

**Published:** 2017-02-02

**Authors:** Yansong Lin, Chen Wang, Wen Gao, Ruixue Cui, Jun Liang

**Affiliations:** ^1^ Department of Nuclear Medicine, Peking Union Medical College Hospital, Beijing 100730, China; ^2^ Department of Oncology, The Affiliated Hospital of Qingdao University, Qingdao 266555, China; ^3^ Department of Oncology, Peking University International Hospital, Beijing 102206, China

**Keywords:** apatinib, radioiodine refractory differentiated thyroid cancer, thyroglobulin, maximum standard uptake value, safety

## Abstract

Currently, patients with radioiodine refractory differentiated thyroid cancer (RAIR-DTC) have limited treatment options. In this study, we aimed to assess the short-term efficacy and safety of apatinib in RAIR-DTC. Ten adult patients were prospectively enrolled to receive oral apatinib (750 mg q.d). The primary endpoints were change in serum thyroglobulin (Tg) concentration, disease control rate (DCR) and objective response rate (ORR) based on RECIST 1.1 criteria. The secondary endpoints included change in glucose metabolism, evaluated by maximum standard uptake value (SUVmax), and safety. As early as 2 weeks after apatinib treatment, the serum Tg concentration decreased by 21.0% in 8 patients available for detection without interference, and a further sharp decline by 81.4% compared with the baseline level occurred at 8 weeks post-treatment. The DCR and ORR were 100% (10/10) and 90% (9/10), respectively. The sum of tumor diameter shrank to 22.8±8.1 mm from 38.8±15.7 mm (*P*=0.001). Moreover, a significant decrease in SUVmax was observed from 6.53±5.14 to 2.56±1.67 and 2.45±1.48 at 4-week and 8-week time-points after treatment (*P*=0.032 and 0.020), respectively. The common grade 3 adverse events (AEs) included hand-foot-skin reaction (50%), hypertension (30%), and hypocalcemia (20%). No severe AE related to apatinib was observed during treatment. Hence, apatinib seems to be a promising therapeutic option for RAIR-DTC patients. Apart from RECIST 1.1 criteria, the biochemical marker (Tg) and glucose metabolism index (SUVmax) could be adopted in assessing the early response to TKI in RAIR-DTC.

## INTRODUCTION

Differentiated thyroid cancer (DTC) arises from aberrant follicular cells. It accounts for nearly 95% of all thyroid neoplasm, with papillary thyroid cancer (PTC) comprising 90% of the DTC [1]. With a rapidly rising incidence especially of PTC in recent decades, DTC has become a global concern [2, 3]. Usually, most patients with DTC could achieve good prognoses following surgery, radioactive iodine (RAI) therapy, or thyroxin therapy [4]. However, 1–23% of DTC patients unfortunately develop distant metastases, 30% of whom will progress to radioiodine refractory DTC (RAIR-DTC) [5, 6]. With a 10-year survival rate less than 10%, RAIR-DTC is the major cause of thyroid cancer-related death [6, 7]. Recent discoveries in the molecular mechanisms implicated in RAIR-DTC have provided insight of its pathogenesis and progression [8, 9]. For example, BRAF^V600E^ mutation has been reported to be associated with non-radioiodine-avid status in distant metastatic lesions, and might account for the mechanism of RAIR-PTC [10]. Besides, a range of 39.5% to 100% of vascular endothelial growth factor (VEGF) expression also indicated the possible implication of VEGF and its related pathway in the course of DTC gradually becoming refractory to RAI therapy [11–13]. Our earlier study using ^99m^Tc-3PRGD2 targeting integrinαvβ3 imaging further indicated an *in-vivo* up-regulation of angiogenesis in lesions of RAIR-DTC [14]. All these findings provided evidences for the potential indication of VEGF receptor (VEGFR) inhibitor in RAIR-DTC [15].

Apatinib is a small-molecule tyrosine kinase inhibitor (TKI) that highly and selectively inhibits VEGFR-2, leading to inhibition of VEGF-mediated endothelial cell migration and proliferation and decrease in tumor microvascular density. Previous studies have proven the potent efficacy and acceptable safety profile of apatinib in gastric cancer [16, 17]. However, there has been no report regarding its use in thyroid cancer so far. This pilot study firstly focused on the early efficacy evaluation of apatinib in RAIR-DTC. Along with the response evaluation in terms of Response Evaluation Criteria in Solid Tumors (RECIST), thyroglobulin (Tg) monitoring and positron emission tomography / computed tomography (PET/CT) scan were also applied to identify the early biochemical and lesion's glucose metabolic response to apatinib.

## RESULTS

### Patient

**Figure 1:**
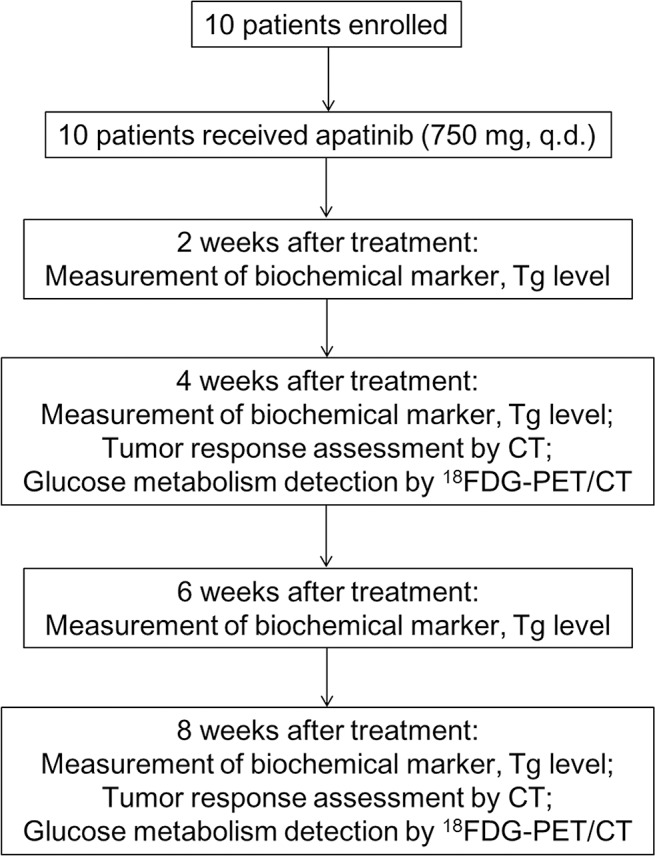
Study diagram

A total of 10 patients were included in this study (Figure 1), aged from 32 to 76 years with a mean of 54.9 years. The male-to-female ratio was 1:1. All patients underwent total or near-total thyroidectomy and lymph node (LN) resection and were pathologically confirmed as PTC. Nine of the 10 patients lost the ability of RAI uptake in metastatic lesions at the first RAI treatment after successful remnant ablation. The Tg levels ranged from 0.43 to 7591 ng/mL at baseline. All participants received apatinib treatment and thus were included in the efficacy and safety analyses. All of the target lesions met the RECIST 1.1 criteria were pulmonary metastatic lesions. The baseline characteristics of the enrolled patients are shown in Table 1.

**Table 1:** The baseline characteristics of enrolled patients and tumor response 8 weeks after apatinib treatment

No.	Gender	Age	Pathology	TNM stage	Metastasis site	VEGF	Accumulated RAI dose (mCi)	RAI* uptake	Response
1	M	44	PTC	pT3N1bM1	Pulmonary & head	-	300	No	PR
2	M	47	PTC	pTxN1aM1	Pulmonary & bone	-	305	No	PR
3	M	76	PTC	pT4aN1bM1	Pulmonary	+	300	No	PR
4	F	32	PTC	pT4aN0M1	Pulmonary	-	200	No	SD
5	M	51	Poorly-PTC	pT4aNxM1	Pulmonary	+	350	No	PR
6	F	75	PTC	pT4aN1bM1	Pulmonary & bone	-	230	No	PR
7	M	65	PTC	pT4aN1bM1	Pulmonary	N/A	350	No	PR
8	F	46	PTC	pT3N1bM1	Pulmonary & bone	-	630	No	PR
9	F	63	PTC	pT4aN1bM1	Pulmonary	+	325	Yes	PR
10	F	49	PTC	pT4aN1bM1	Pulmonary	-	440	No	PR

VEGF: vascular endothelial growth factor; RAI: radioactive iodine; PTC: papillary thyroid cancer; PR: partial response;

SD: stable disease;.

N/A: not available.

*RAI uptake in metastasis lesions at the first RAI therapy.

### Efficacy

#### Evaluation of early biochemical response

**Figure 2:**
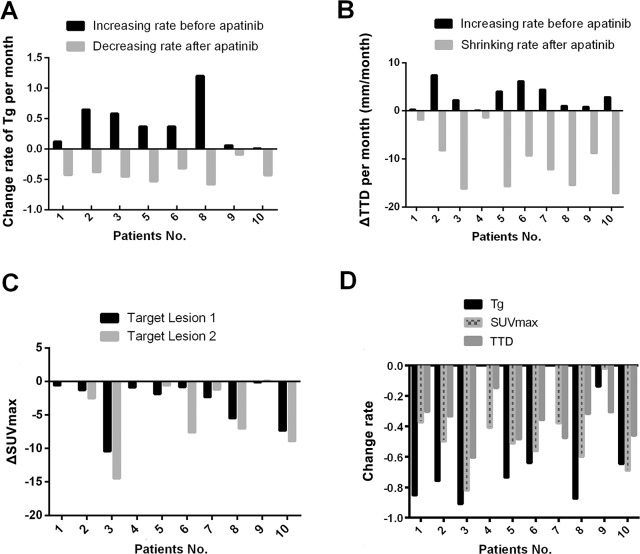
The changes in Tg, TTD, and SUVmax before and 8 weeks after apatinib therapy **A**. The change rate of Tg per month in individual patients before and after apatinib therapy. The change rate of Tg per month was calculated according to the following formula: (Tg–Tg_baseline_)/ Tg_baseline_/ month. **B**. The change in sum of target lesion diameter per month in individual patients before and after apatinib therapy. The change in sum of target lesion diameter per month (ΔTTD per month) was calculated according to the following formula: (TTD–TTD_baseline_)/month. **C**. The change in SUVmax of target lesions after apatinib therapy. The change in SUVmax was defined as ΔSUVmax, which was calculated according to the following formula: SUVmax–SUVmax_baseline_. **D**. The change rate of Tg, SUVmax, TTD in individual patient after 8 weeks of apatinib therapy. The change rate of Tg was calculated according to the following formula: (Tg–Tg_baseline_)/ Tg_baseline_. The change rate of SUVmax was calculated according to the following formula: (SUVmax–SUVmax_baseline_)/SUVmax_baseline_. The change rate of TTD was calculated according to the following formula: (TTD–TTD_baseline_)/TTD_baseline_. Tg, thyroglobulin; TTD, total tumor diameter which was defined as sum of the diameter of target lesions; SUVmax, maximum standard uptake value.

**Figure 3:**
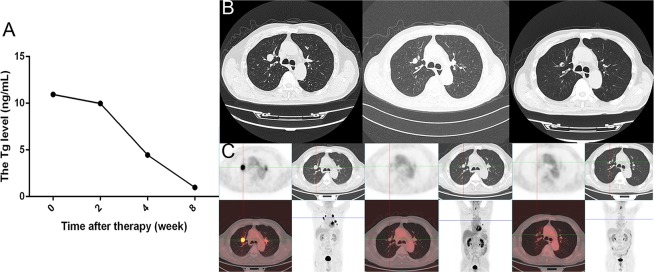
The Tg, CT, and PET/CT results obtained before and after apatinib therapy in a male patient **A**. Tg levels at baseline and after therapy. **B**. CT images at baseline and after 4 and 8 weeks of apatinib therapy. **C**. PET/CT images at baseline and after 4 and 8 weeks of apatinib therapy. Tg, thyroglobulin; CT, computed tomography; PET/CT, positron emission tomography/ computed tomography.

Eight out of the 10 patients were available for early biochemical response evaluation, while the remaining 2 patients were excluded from analysis due to interference with high-level thyroglobulin antibody (TgAb) (>4000 IU/mL) in Tg detection or low levels of Tg at baseline and during treatment (<1 ng/mL). The Tg concentration increased by 41.0% within the 12 months before apatinib therapy, but decreased by 40.2% after apatinib administration (the individual responses are shown in Figure 2A). In fact, as early as 2 weeks after therapy, a decline by 21.0% in Tg could be noticed, indicating a rapid biochemical response to apatinib. Furthermore, the Tg concentration was continually decreased by 81.4% from the baseline level (from 1583.5 ng/mL at baseline to 294.6 ng/mL at 8-week time-point). Figure 3A shows a sharp decline in Tg level in a 76-year-old male patient. Unlike other cases, one 46-year-old female patient did present an increase in Tg level from 7591.0 ng/mL to 11191.0 ng/mL 2 weeks after therapy; however, the Tg level decreased to 2947.0 ng/mL 4 weeks after treatment.

#### Evaluation of target lesions response according to RECIST 1.1

Eighteen target lesions of the 10 patients were identified and evaluated. The growth rate was 1.38 ± 0.98 mm per month within the 12 months before apatinib treatment, and the total tumor diameter (TTD, defined as sum of the diameter of target lesions) was 38.8 ± 15.7 mm at baseline. CT results indicated that 40.0% of early tumor shrinkage was achieved 4 weeks after apatinib administration, with the TTD diminishing from 44.7 ± 11.0 to 29.6 ± 8.0 mm (*P*<0.001) in 7 patients available for evaluation. Eight weeks later, the TTD shrank down to 22.8 ± 8.1 mm (*P*=0.001; the individual responses are shown in Figure 2B). Figure 3B shows a target lesion of the 76-year-old male patient, the diameter of which decreased from 21.3 mm to 15.9 mm and 9.5 mm after 4 and 8 weeks of treatment, respectively.

Eight weeks following apatinib administration, 9 patients achieved partial response (PR) and one patient achieved stable disease (SD), according to the RECIST 1.1 criteria (Table 1). The disease control rate (DCR) and objective response rate (ORR) of the study were 100% (10/10) and 90% (9/10), respectively.

#### Evaluation of glucose metabolic response of target lesions based on PET/CT

Significant decrease in maximum standard uptake value (SUVmax) was revealed after 8 weeks of treatment, indicating a rapid decrease in glucose metabolism in the target lesions. The SUVmax diminished from 6.53 ± 5.14 to 2.56 ± 1.67 (*P*=0.032) and 2.45 ± 1.48 (*P*=0.020) after 4 and 8 weeks of treatment, respectively (the responses of each lesion are shown in Figure 2C). Especially, in the male patient mentioned above, the SUVmax decreased from 11.38 to 1.26 and 0.95 at the 4- and 8-week time-point following apatinib treatment, respectively (Figure 3C).

#### The correlation among Tg, lesions diameter and SUVmax

**Figure 4:**
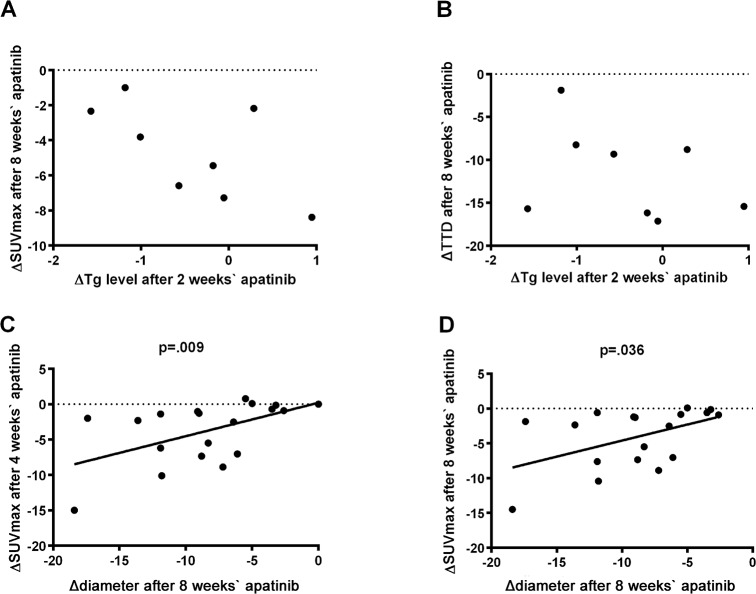
The association between Tg, TTD and SUVmax **A**. Relationship between changes in SUVmax after 8 weeks of apatinib therapy and Tg after 2 weeks of apatinib therapy in patients. **B**. Relationship between changes in TTD after 8 weeks of apatinib therapy and Tg after 2 weeks of apatinib therapy in patients. **C**. Relationship between changes in SUVmax after 4 weeks of apatinib therapy and diameter after 8 weeks of apatinib therapy in target lesions. **D**. Relationship between changes in SUVmax after 8 weeks of apatinib therapy and diameter after 8 weeks of apatinib therapy in target lesions. ΔTg: Tg–Tg_baseline_; ΔTTD: TTD–TTD_baseline_; Δdiameter: diameter-diameter_baseline_; ΔSUVmax: SUVmax–SUVmax_baseline_. Tg, thyroglobulin; TTD, total tumor diameter which was defined as sum of the diameter of target lesions; SUVmax, maximum standard uptake value.

The decrease rate of Tg level, TTD and SUVmax 8 weeks after apatinib administration were compared within individual cases (Figure 2D). Tg exhibited the most and fastest decrease, followed by glucose metabolism revealed by PET/CT and TTD reflected by CT. The relationships among changes in the three indicators were further analyzed. No correlation was found in patients between the changes in Tg level and SUVmax (after 8 weeks) or Tg level and TTD (Figure 4A, 4B), while linear correlations of change in diameter after 8 weeks of therapy with changes in SUVmax after 4 and 8 weeks of therapy were observed in the target lesions (*P*=0.009 and 0.036, respectively) (Figure 4C, 4D).

### Safety

In this study, most patients suffered from grade 1 to grade 3 treatment-related adverse events (AEs), and no severe AE (SAE) related to apatinib was observed during the 8 weeks treatment period. The most common AE was hand-foot-skin reaction (HFSR) (9/10). No cardiovascular AE was reported. The grade 3 AEs included HFSR, hypertension, hypocalcemia, proteinuria, neutropenia, thrombocytopenia, pharyngolaryngeal pain, and dysphagia, which accounted for 50%, 30%, 20%, 10%, 10%, 10%, 10%, and 10%, respectively. Treatment suspension was required due to grade 3 HFSR, proteinuria, neutropenia, thrombocytopenia, or dysphagia. The details in AEs related to apatinib are shown in Table 2, with a comparison with those of sorafenib and lenvatinib as previously described [1, 7]. AEs were listed if they were reported in more than 10% of patients.

**Table 2:** Adverse events

Adverse events (%)	Apatinib		Lenvatinib [7]		Sorafenib [1]	
	All grades	Grade≥3	All Grades	Grade≥3	All Grades	Grade≥3
HFSR	90	50	31.8	3.4	76.3	20.3
Hypertension	80	30	67.8	41.8	40.6	9.7
Proteinuria	70	10	31.0	10	NA	NA
Elevated transaminase	70	0	NA	NA	12.6/11.1	2.9/1.0
Fatigue	70	0	59.0	9.2	49.8	5.8
Hypocalcemia	70	20	6.9	2.7	18.8	5.8
Hyperbilirubinemia	40	0	NA	NA	NA	NA
Dyspepsia	40	0	10	0	31.9	2.4
Mucositis oral	40	0	35.6	4.2	23.2	0.5
Dysgensia	40	0	16.9	0	NA	NA
Leukopenia	30	0	NA	NA	NA	NA
Neutropenia	30	10	NA	NA	NA	NA
Elevated GGT	20	0	NA	NA	NA	NA
Thrombocytopenia	20	10	NA	NA	NA	NA
Pharyngolaryngeal pain	20	10	10	0.4	10.1	0
Dysphagia	20	10	NA	NA	NA	NA

HFSR: hand-foot-skin reaction; GGT: gamma-glutamyl transpeptidase.

N/A: not available.

## DISCUSSION

Progress has been made in revealing the molecular mechanisms of thyroid cancer in the past 10 years [18], thus promoting clinical translational research in RAIR-DTC. The enhanced catalytic activity via the phosphorylation of many intracellular proteins involved in the signal transduction cascade is responsible for uncontrolled cell growth and thus is the molecular rationale for the use of TKIs in thyroid cancer. Several TKIs have been tested for the treatment of advanced, progressive DTC patient who no longer benefits from radioiodine therapy, with a PR rate varying from 8% to 64.7% [1, 7, 19–22]. Sorafenib is the first FDA-approved TKI, which targets VEGFR 1, 2, and 3, platelet-derived growth factor receptor (PDGFR), Raf-1, RET, and BRAF. It displayed a PR rate of 12.2%. Lenvatinib, a TKI targeting VEGFRs, fibroblast growth factor receptors, and PDGFR, was the second TKI approved for its clinical use in RAIR-DTC. Lenvatinib achieved a reported PR rate of 64.7%. In this pilot study, PR was achieved in 90% of patients after only 8 weeks of apatinib administration. Relatively, this is the best and fast PR rate, which had ever achieved in RAIR-DTC treated with TKIs. This rapid response also shed light on using apatinib as pre-operative adjuvant therapy, which aims at quickly reducing the tumor burden and facilitating complete tumor resection. Though it remains unclear, the highly specific and selective inhibition of VEGFR-2 by apatinib potently represses VEGF-mediated endothelial cell migration and proliferation, which might be responsible for the majority of its high efficacy [23]. *In vitro*, enzyme experiments showed that apatinib (YN968D1) was a highly selective inhibitor of VEGFR-2 with an IC50 of 1 nM. The value is much lower than that of sunitinib (5 nM), sorafenib (90 nM) or even lenvatinib (4 nM), indicating that apatinib can effectively inhibit the proliferation, migration and tube formation of human umbilical vein endothelial cell [23–25]. Besides, the difference between Chinese and Western population concerning genomic and demographic characteristics might also play a role [26–28].

The challenges to assess the response to TKI include finding suitable markers to reflect its early efficacy and timely identifying the patients who are unlikely to benefit from expensive TKI therapy that is not covered by Chinese health insurance and cannot be afforded by most of the Chinese families. Fortunately, Tg, an organ-specific glycoprotein that is produced only by normal thyroid or differentiated thyroid cancer cells, becomes a sensitive marker for the surveillance of thyroid cancer. Serum Tg was proven to be a specific marker for predicting distant metastases after surgery even without any imaging evidence before radioiodine ablation therapy in our previous work [29]. Also, it becomes a reliable marker for detecting DTC recurrence after successful remnant thyroid ablation [30, 31]. All patients enrolled in this study achieved a successful thyroid remnant ablation after at least one time of radioiodine therapy. We noticed an immediate reduction of Tg level as early as 2 weeks after apatinib therapy, and a more pronounced Tg reduction can be observed compared with the simultaneous changes in TTD and glucose metabolism, implying that Tg could be used as both sensitive and convenient serum indicator in reflecting the early biochemical response to TKI in RAIR-DTC. One patient presented a transient increase in Tg after 2 weeks of therapy but a dramatic decrease 4 weeks post-treatment, which may be caused by the probable Tg release due to the self-destruction of tumor cells in response to apatinib [32]. Considering this early change in Tg, we proposed that RECIST evaluation, which was indicated at least 2 to 3 months after TKI in most previous studies [19–22], might miss the probable early anatomic response in RAIR-DTC. CT evaluation at 4-week time-point in this study did identify more than 40.0% of initial mean shrinkage, which indicated an early partial response in 14 target lesions available for assessment. Thus for the first time, we demonstrated that RECIST assessment could be applied earlier than currently recommended for the purpose of early response identification. In addition, anatomic imaging alone using RECIST1.1 response criterion has the limitation in reflecting metabolism. Over recent years, there is an evolving consideration for ^18^FDG-PET assessment in solid tumors, which is gradually accepted as PERCIST 1.0 response criteria and proven to be a complementary assessment method in both clinical trials and structural quantitative clinical evaluation [33, 34]. Our study substantiated a dramatic decrease in SUVmax after 4 weeks of therapy using ^18^FDG-PET/CT, which was correlated to the decrease in target lesion diameter revealed by CT. Besides, more significant decrease in SUVmax was observed compared with the simultaneous structural change reflected by RECIST (60.8 % vs 34.0%), implying an earlier and more pronounced metabolic response prior to structural reaction in response to apatinib. Thus ^18^FDG PET holds the promise to afford an on-going *in-vivo* biochemical evidence for the early assessment of response to TKI in RAIR-DTC. Would an earlier ^18^FDG PET be more informative for the treatment response assessment? Several studies demonstrated the promising application of ^18^FDG PET in the early evaluation of lung cancer treated with epidermal growth factor receptor (EGFR) TKI, with varied evaluation time, even as early as 2 days after drug administration [35, 36]. For thyroid cancer, researchers ever tried to perform the PET/CT after 7 days treated with sunitinib, but failed to capture its significant therapeutic effect [21]. This might be due to the different mechanism involved in TKI treatment. Early tumor necrosis suggested by the variation of Tg after 2 weeks of apatinib in this study might also be a confounding factor, which prevents an interpretation of an earlier PET evaluation less than 2 weeks. Thus, considering the influence factors on efficacy assessment, radiation exposure and safety, PET/CT at 4 weeks set in this study made sense and came out with both encouraging and convincing results. Significant falling of Tg achieved PR after 8 weeks of therapy, also indicating that monitoring Tg level could be a handy and useful method to detect the early response to TKI in RAIR-DTC. Our study showed no significant correlation between changes in Tg and SUVmax or TTD, yet, the possible reasons might be the limited numbers of cases, or the dedifferentiation of tumor partially losing the ability to produce Tg. In a word, the early evaluation modalities we adopted in this study revealed that the 4 weeks RECIST evaluation combined with Tg and glucose metabolism detection might be helpful in earlier identifying patients who unlikely response to those expensive and toxicity TKI and should withdraw from the unbeneficial therapy as early as possible.

In addition to the quick response and high efficacy of apatinib, acceptable and predictable toxicities were observed [16, 17]. HFSR was the most frequent AE during the therapy. As the most common AE of TKI, despite not life threatening, HFSR might seriously impair activities and daily life, even causing drug suspending and reduction [37]. In this study, 90.0% patients presented with HFSR, compared to 31.8% of lenvatinib and 76.3% of sorafenib in treating thyroid cancer [1, 7]. Five of them (50.0%) suffered from grade 3 HFSR (3.4 % of lenvatinib and 20.3% of sorafenib), resulting in therapy suspension at the third to the fifth week [1, 7]. After drug discontinuation for one week, the HFSR receded to grade 1. All AEs were controllable and most of them were well tolerated. Though tolerable, patients with thyroid cancer seemed to experience more HFSR and grade 3 AEs compared with Li's phase III study in patients with advanced gastric cancer, who might present malabsorption by gastric surgery or by digestive tumor itself, implying that dose adjustment might be needed to balance the efficacy and adverse effect when treating RAIR-DTC [17].

To summarize, our results presented safe, rapid and high efficacy of apatinib in patients with RAIR-DTC. Along with RECIST evaluation, Tg and ^18^FDG-PET/CT could afford more informative and distinct biochemical and glucose metabolism changes, and could be used as early response indicators. Further follow-up of this study are still underway, and the overall survival (OS), progression free survival (PFS), duration of response (DoR) will be reported.

## PATIENTS AND METHODS

This study was approved by the institutional review board of Peking Medical College Hospital Ethics Committee, and was conducted in accordance with the Declaration of Helsinki and International Conference on Harmonization Good Clinical Practice guidelines. The informed consents were obtained from all patients before enrollment. This study is registered with ClinicalTrails.gov (number, NCT02731352).

### Patients

Patients aged 18 years or older with histologically confirmed locally advanced or metastatic RAIR-DTC were recruited in this study. The inclusion criteria included at least one progressive lesion according to the RECIST (version 1.1); disease progression within the past 14 months according to RECIST (version 1.1); and meet the following definition of RAIR-DTC. Patients were classified as RAIR-DTC if they met one of the following criteria: 1) had at least one measurable lesion without iodine uptake on ^131^I scan, 2) had one measurable lesion that had progressed within the past 12 months even it could uptake radioiodine, or 3) received cumulative activity of ^131^I over 600 mCi. All eligible patients had received no prior therapy with a TKI or chemotherapy.

### Study design and treatment

Both efficacy and safety of apatinib (Jiangsu Hengrui Medicine, Lianyungang, China) were evaluated in this study. The study diagram is shown in Figure 1. All enrolled RAIR-DTC patients were commenced on apatinib at a dose of 750 mg q.d. A treatment cycle was defined as 4 weeks. Treatment interruption caused by toxicities of apatinib was allowed for no more than 2 times or a maximum duration of 2 weeks (either continuously or cumulatively) in one cycle. Dose reduction to 500 mg q.d. was accepted for only one time, and re-escalation was not accepted. Treatment continued until the disease progression, drug intolerance or withdrawal of consent from the study.

The primary endpoints were change in serum Tg concentration, DCR and ORR. The secondary endpoints included OS, PFS and DoR, changes in iodine metabolism, glucose metabolism and tumor angiogenesis, and safety. The present report described the early response of apatinib in RAIR-DTC, including change in Tg level, DCR, ORR, change in glucose metabolism in target lesions, and safety during 2 treatment cycles.

### Study assessments

VEGF expression in the primary foci was detected by immunohistochemistry using Vectastain ABC kit (Vector, Burlingame, USA). After pretreatment and blocking, deparaffinized section (3-mm) was incubated with primary antibody (Abcam Inc., Cambridge, USA) at 4°C overnight followed by secondary antibody at room temperature for 30 min. The results were analyzed by two independent pathologists.

The serum Tg concentrations at baseline and every 2 weeks after treatment during the therapy were measured. Simultaneously, the TgAb was detected to identify potential assay interference. Measurements were performed by electrochemiluminescence immunoassay (Roche Diagnostics GmbH, Mannheim, Germany) with a functional sensitivity of 0.100 ng/mL and 10 IU/mL, respectively. Change in Tg was defined as ΔTg, which was calculated as Tg-Tg_baseline_. The change rate of Tg was calculated as (Tg-Tg_baseline_)/Tg_baseline_, and change rate per month was also calculated.

Tumor diameters were measured at baseline and every 4 weeks after treatment using CT. TTD was defined as the sum of the tumor diameter of each lesion. Change in TTD (ΔTTD) per month was calculated as (TTD–TTD_baseline_)/month, and change rate of TTD was calculated as (TTD–TTD_baseline_)/TTD_baseline_. Furthermore, tumor response was evaluated as defined by RECIST (version 1.1).

Additionally, ^18^F-fluorodeoxyglucose (^18^FDG) PET/CT scan was conducted at baseline and every 4 weeks after treatment to assess the glucose metabolic response to apatinib. Briefly, patients were instructed to avoid strenuous work or exercise for at least 24 hours and fast for more than 4 hours before the ^18^F-FDG (0.15 mCi per kilogram) injection. After injection, patients were rested in a warm, darkened room for nearly 40 minutes. The acquisition was performed from skull to mid-thigh (5 to 6 beds position, 2 min per bed) using a Siemens Biograph 64 Truepoint TrueV PET/CT scanner. Images were reconstructed with manufacturer-provided software by using an ordered subset expectation maximization algorithm. SUVmax was used to evaluate the ^18^F-FDG uptake in a lesion. Change in SUVmax was defined as ΔSUVmax, which was calculated as SUVmax–SUVmax_baseline_. The change rate of SUVmax was calculated as (SUVmax–SUVmax_baseline_)/ SUVmax_baseline_,

Adverse events were assessed and recorded until 4 weeks after the last drug of the study was administered.

### Statistical analysis

Categorical variables were expressed as number and percentage, and continuous variables were expressed as mean ± standard deviation (SD) or median and interquartile range when appropriate. A two-sample t-test or Wilcoxon rank sum test was used to compare the tumor diameter before and after apatinib treatment. Correlation between two variables was analyzed by linear regression. SPSS (version 19.0; SPSS Inc, Chicago, IL, USA) and Prism 6 (GraphPad Software, San Diego, CA, USA) were used for statistical analyses. A *P* value less than 0.05 was considered statistically significant.
